# Incremental Encoder Speed Acquisition Using an STM32 Microcontroller and NI ELVIS

**DOI:** 10.3390/s22145127

**Published:** 2022-07-08

**Authors:** Adrian Augustin Pop

**Affiliations:** Department of Electrical Machines and Drives, Faculty of Electrical Engineering, Technical University of Cluj Napoca, 400114 Cluj-Napoca, Romania; augustin.pop@emd.utcluj.ro

**Keywords:** microcontrollers, data acquisition, variable speed drives, optical sensors, signal processing algorithms

## Abstract

Precise motor control requires high accuracy of the rotor position through the incremental encoder. The speed and accuracy of the acquisition equipment (microcontroller) play an important element in terms of cost and efficiency. In this paper, the author presents alternative methods for speed acquisition from an incremental encoder. In the first stage of research, the main performances of the STM32 microcontroller, connected with an incremental encoder, will be analyzed and compared with two different acquisition systems, i.e., ELVIS II and a Unidrive M701 power inverter. Using the LabVIEW graphical programming language, a user-friendly, convenient, and flexible human–machine interface is designed. Due to the advantages provided by the STM32 microcontroller in terms of processing power, cost, and programming interface, the obtained results are accurate and consistent. Through experimental testing and analysis, the speed acquisition is stable for both developed software algorithms used for ELVIS II and STM32 platforms. It is the aim of the paper to propose a useful speed acquisition tool in low-cost, high-accuracy prototyping applications.

## 1. Introduction

The industry and researchers need to reduce the time consumed with software development, errors, and costs whenever new products are produced. Working efficiently is indispensable to success in a globalized market, especially for electronic industries such as electric motors manufacturers, power converters, sensors, etc. In new product development, the monitoring and data acquisition systems are often necessary to observe and analyze the characteristics and stability of the used equipment. Many mechanical systems incorporate rotating machinery, and, thus, it is very important to monitor and measure the speed with a high level of precision. In precision systems such as automotive ones, the error in speed measurement can be unacceptable. As such, it is crucial to use high-accuracy data acquisition systems to monitor the speed correctly. Data acquisition is the process of sampling and recording the physical characteristics of systems. These characteristics, known as variables, allow the system to be analyzed. A data acquisition system is an instrument used to measure and record these variables. There are multiple methods of motor speed measurement—see [1,2,3,4,5,6,7]—and different speed estimation methods, as seen in [8,9,10,11]. The authors of [12,13,14] use the FPGA implementation. In this paper, incremental encoders connected with an STM microcontroller are used together with (NI) ELVIS acquisition equipment, and a power inverter. Specifically, the paper proposes original, step-by-step methods to implement the velocity acquisition process with the aid of the STM32 microcontroller. The main advantage is the fast and direct calculation of time derivatives, low power consumption, rapid prototyping, and low cost.

Main differences between the FPGA and STM32, together with the speed measurements methods, will be discussed in detail. The main contributions of the paper are the following:

- State of the art of the newest speed acquisition methods from incremental encoders.
- Setting the main differences between FPGA and STM 32 for speed measurement applications.
- Software implementation on STM 32 and NI ELVIS II.
- Test bench measurement of the speed from an incremental encoder.

The paper is structured as follows. Section 2 presents the frequency and time-period measurement techniques used for speed acquisition from the incremental encoder. The test bench and incremental encoder used in the study are described in Section 3. Section 4 details the STM 32 microcontroller and the code implementation. NI ELVIS is introduced in Section 5 for the measurement and data acquisition integration. In Section 6, experimental results are provided, and some conclusions are drawn.

## 2. Frequency and Time-Period Measurement

### 2.1. Frequency Measurement

The common frequency measurement method for rotor speed acquisition is counting the encoder pulses inside of a time window. This is the simplest speed estimation approach and is also called the M-method. Unfortunately, the M-method is noisy, especially for high frequency. The noisy output is due to the spatial position quantization.

Angular speed is approximated to an incremental ratio in the following equation:

(1) $$\omega =\frac{d\theta }{\mathrm{dt}}≅\frac{∆\theta }{t}≅\frac{2\pi \cdot \Delta n}{N_{p}\cdot t}\left[{\mathrm{rad}\;s}^{-1}\right]\to \frac{60\cdot \Delta n}{N_{p}\cdot t}\left[\mathrm{RPM}\right]\;$$

where N_p_ defines number of pulses per revolution, Δn is the number of detected pulses, and t is the time window.

The quantization error is superimposed on the speed inside the detection time window, which depends on the uncertainty of the number of pulses, Δn. The quantization error, Δω, is:

(2) $$∆\omega =\frac{2\pi }{N_{p}t}\left[{\mathrm{rad}\;s}^{-1}\right]\to \frac{60}{N_{p}t}\left[\mathrm{RPM}\right]\;$$

From Equation (2), the quantization error is influenced by the number of encoder pulses and the detection time window. The precision of this method is speed-dependent:

(3) $$e_{\left(\omega \right)}\%=\frac{2\pi }{\omega \cdot N_{p}t}\cdot 100\%\;$$

The product, $N_{p}t$, is constant for high- and low-resolution encoders. The absolute measurement error is not influenced by the speed values, as shown by Equation (2). The frequency measuring method is very simple, requiring the computation of ∆n and one multiplication for constant terms in Equation (1).

The quadrature decoder hardware is used for incremental position measurement, and it can be found inside microcontrollers. If a low pass filter is used, the implementation of an nth-order filter is very simple but is time-consuming if the higher order is considered.

### 2.2. Time Period Measurement

This method consists of measuring the time period between two encoder pulses using high-frequency clock pulses. The time period is measured with the aid of a central processing unit timer of the microcontroller; therefore, shorter time periods are reflected in a higher speed. To reduce the measurement error, an alternative solution is to change the frequency measurement to a period measurement at a given speed.

**Figure 1:**
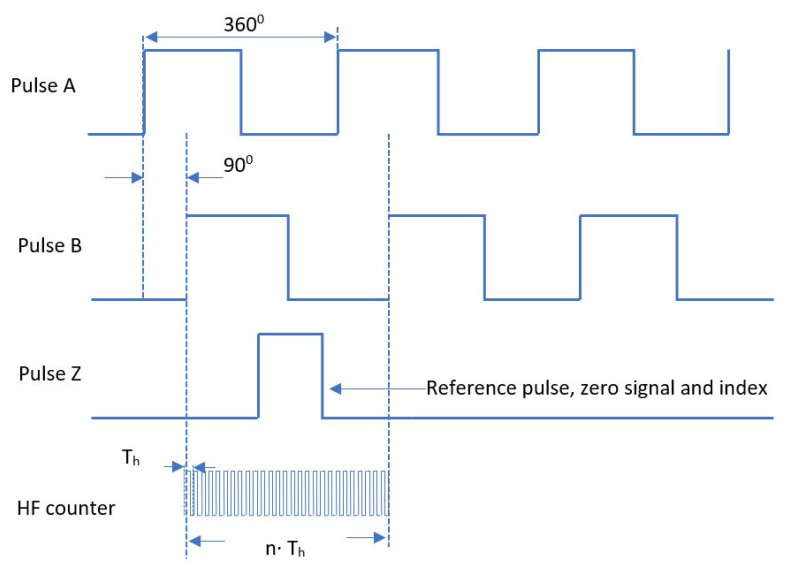
Incremental encoder. The period measurement by a high-frequency counter.

The measurement is simple and involves measuring the number of periods of a high-frequency signal generated by encoder signals, Figure 1.

The Equation (4) is valid only if the motor speed is constant:

(4) $$\omega =\frac{d\theta }{\mathrm{dt}}≅\frac{\Delta \theta }{n\cdot T_{h}}≅\frac{2\pi }{N_{p}\cdot n\cdot T_{h}}[\mathrm{rad}\cdot s^{-1}]\to \frac{60}{N_{p}\cdot n\cdot T_{h}}\;\left[\mathrm{RPM}\right]$$

The speed sampling period is proportional to the motor speed:

(5) $$T_{\left(\omega \right)}\left(\omega \right)=\frac{2\pi }{N_{p}\cdot \omega }\left[s\right]$$

The measurement’s precision is determined by the ratio between the period of the encoder signal and the high frequency counter. The absolute error of a high-frequency pulse will be represented in the equation below, where $T_{h}$ is the time period corresponding to high frequencies:

(6) $$e_{\omega }\%=\frac{T_{h}}{\frac{2\pi }{N_{p}\cdot \omega }-T_{h}}\cdot 100\%≅\frac{\omega \cdot N_{p}\cdot T_{h}}{2\pi }\;$$

When running the motor at a very low speed, the number of pulses (n) can be very high, so the timer from microcontroller (digital timer) can saturate.

Implementing the period measuring method requires a timer capture unit, which can be found, nowadays, in microcontrollers such as STM32. To overcome digital timer saturation, hardware subsystems can be considered. An adapted software algorithm to filter the signals and eliminate the errors can also be introduced.

### 2.3. Mixed Mode Frequency and Time Period Measurement

The mixed mode is often adopted in industrial inverters. The need for a specific hardware subsystem (for eliminating the digital saturation) is achieved by using FPGA devices that have progressed to a high level of maturity, and which can be used complementarily to the traditional Application Specific Integrated Circuits (ASICs). Recent FPGAs can work with low-power consumption that enables economical implementation and features parallel operation [12]. Although FPGA may be used for high-performance applications, sophisticated algorithms still represent a significant design challenge [13].

**Figure 2:**
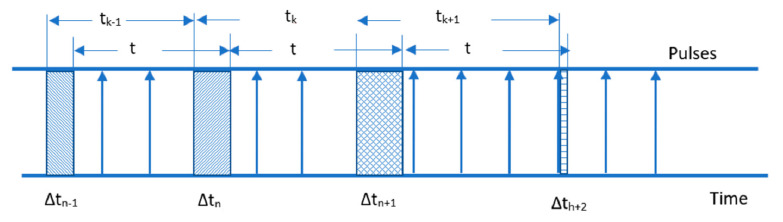
Error compensation using an observation window.

Another approach is described in [14], with low errors. The main idea is to find the quantization error of the frequency. The compensation can be made by measuring the time intervals ($∆t_{n})$ between the limits of the observation window (t) and the encoder pulses, Figure 2. We can now modify the window width ($t_{k})$ at the nearest integer number of pulses, and, therefore, eliminate the quantization error.

The speed can be expressed as follows:

(7) $$\omega =\frac{\Delta N}{\Sigma t+{\Delta t}_{n-1}-{\Delta t}_{n}}\cdot \frac{2\pi }{N_{p}}\;\left[\mathrm{rad}\cdot s^{-1}\right]\to \frac{\Delta N}{\Sigma t+{\Delta t}_{n-1}-{\Delta t}_{n}}\cdot \frac{60}{N_{p}}\;\left[\mathrm{RPM}\right]\;$$

ΔN is the number of encoder pulses in the observation window.

This equation can be used for low and high speed without the need of complicated microcontrollers or FPGA. The measuring error value is given by:

(8) $$e_{\omega }\%=\frac{2\cdot T_{h}}{\Sigma t+{\Delta t}_{n-1}-{\Delta t}_{n}+2\cdot T_{h}}\cdot 100\%\;$$

### 2.4. Divisionless (DT) Algorithm for the MT-Type Speed Estimation Method

**Figure 3:**
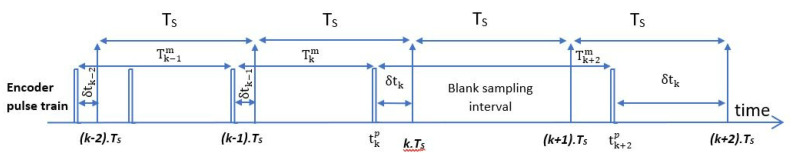
Sampling intervals for speed computation.

This method was introduced in [13]. To read the angular speed from an incremental encoder, there are three methods to do so: M-Type, T-type, and a combination of both MT-type. The M-type is more accurate for high speeds and is suitable for real-time systems. The second one provides highly accurate measurements at low speed and needs more computational time. The third one is a combination of the first two. Here, the authors used a divisionless MT algorithm on an optimized FPGA. The objective was to calculate speed throughout a set of sample intervals, with exact actual position data available at the sampling time instants, Figure 3. The sample period can be seen as the optimal speed information. The disadvantage of this strategy is that incremental encoders, due to the nature of encoder pulses, cannot reveal the real position at the sampling instant (asynchronous).

We can assume that the speed change throughout sampling periods is near zero. As a result, the speed estimation from prior sampling can be used. This method can be summarized as follows [13]:

(9) $${\hat{X}}_{k}^{a}=X_{k}+{\hat{V}}_{k-1}{\delta t}_{k}$$

(10) $${\hat{V}}_{k}=\frac{{\hat{X}}_{k}^{a}-{\hat{X}}_{k-1}^{a}}{T_{s}}$$

where ${\hat{X}}_{k}^{a}$ is the approximated actual position, the time instant is $t_{k}={\mathrm{kT}}_{s}$, and ${\hat{V}}_{k}$ is the estimated velocity. The real position of the sampling instant is estimated by Equations (9) and (10), and it produces a speed prediction. Unfortunately, in the general case, these equations cannot give consistent speed estimation and must be adjusted.

In the generalized scenario, the authors of [13] offer an adaption of the DLMT (divisionless MT) algorithm that provides an asymptotically stable output.

The speed estimate approach for the generalized divisionless algorithm for the MT-type (GDLMT) is given by

(11) $${\hat{X}}_{i}^{a}=X_{i}+{\hat{V}}_{i-1}{\delta t}_{i}$$

(12) $${\hat{V}}_{i}=\frac{{\hat{X}}_{i}^{a}-{\hat{X}}_{i-1}^{a}}{m_{i-1}T_{s}}$$

The proposed GDLMT has a dynamic equilibrium and is equivalent to the output of the MT method. The GDLMT algorithm has the variable term $m_{i-1}T_{s}$, and it implies algorithmic division. However, this solution is proposed to be developed on classic FPGA platforms, being affected by the complexity of the programming level needed for integrating the arithmetical division.

**Figure 4:**
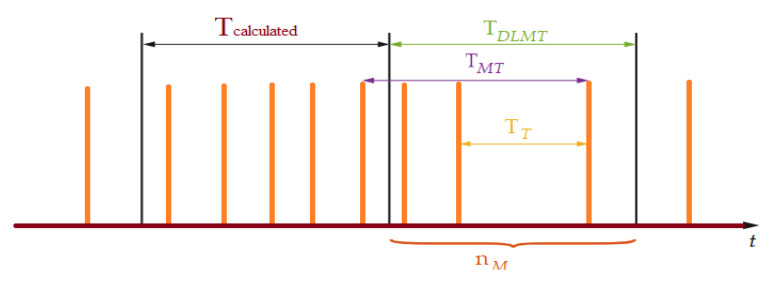
MT and DLMT algorithms of measurement [15].

In Figure 4, the main differences between MT and DLMT are emphasized. T_calculated_ is the calculation period; T_T_ is the time interval between the previous two pulses; n_m_ is the number of pulses on the current interval; T_MT_ is the time interval between the last pulse of the current period and the pulse of the period where at least one pulse existed.

The authors of [15] proposed an angular speed estimation for different calculation frequencies. The encoder’s digital signal is sampled at 1 MHz.

**Figure 5:**
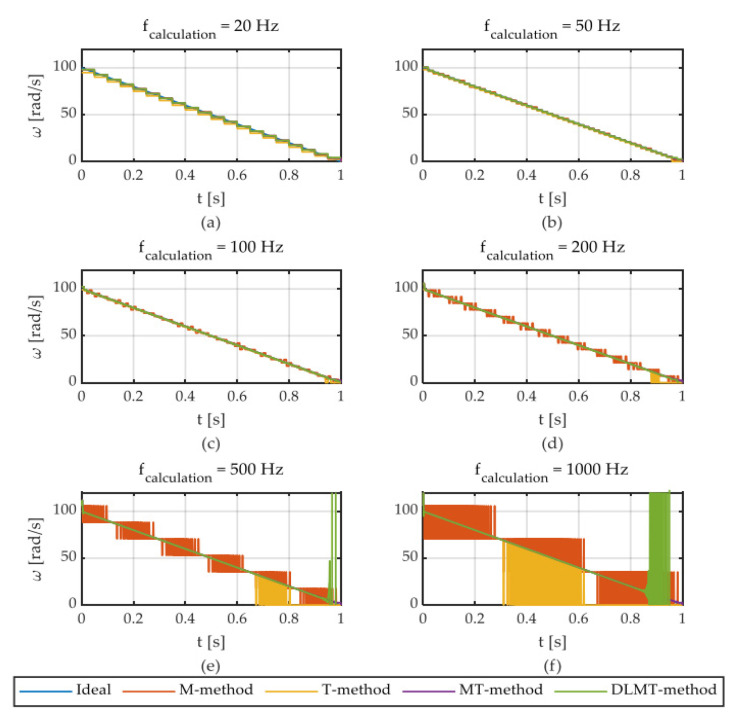
Speed estimation by M, T, MT, and DLMT algorithms for different calculation frequencies [15].

From 20 to 1000 Hz, the angular speed was estimated, Figure 5. The noise is small at a low frequency. The M and T methods add some noise to the signals for higher frequencies. The T method provides good stability above the demanded speed.

The MT technique performs well, but only at very low angular speeds. The MT approach has the drawback of requiring a lot of computing power. The DLMT algorithm provides a similar performance to the MT type, but not at low speeds when significant oscillations can be observed.

For simulation purposes, the frequency of 100 Hz is fixed because it provides a good balance between precision and measurement delay.

**Figure 6:**
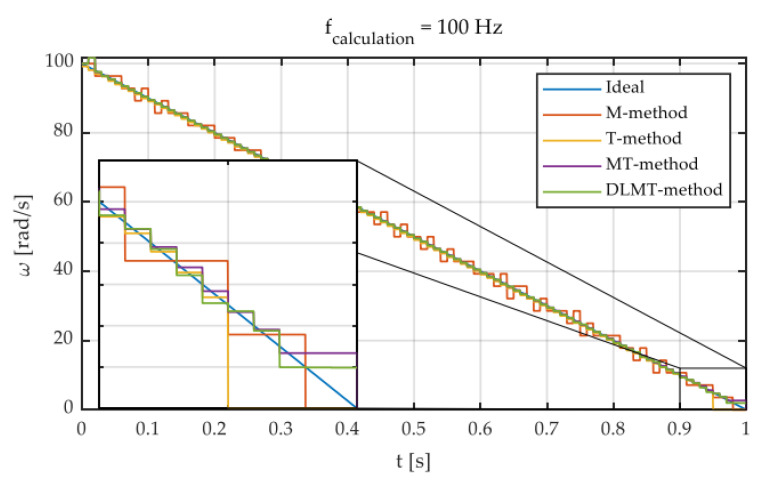
Main differences between M, T, MT, and DLMT speed measurement algorithms [15].

The input speed is given by the linear function ω(t)=100−100t. For higher speeds, the performance of DLMT is comparable to that of MT. The disadvantage is that it cannot provide sufficient measures for low angular speeds. From Figure 6, the main differences between the M, T, MT, and DLMT algorithms can be revealed. Unfortunately, it is not very stable at low angular speeds.

## 3. Speed Measurement with Incremental Encoder

Encoders come in a variety of shapes and sizes. In most cases, incremental encoder sensors are connected for angular speed detection. Resolvers are another type of sensor used to determine absolute mechanical angles. Due to their accuracy, resolution, and robustness, resolvers are widely employed in industrial applications. Resolvers produce sinusoidal electrical signals with respect to the position of the shaft. To calculate the angular position, these signals must be handled with an appropriate converter. To accomplish this objective, open-loop conversion methods are developed in the literature [16,17].

The encoder is equipment used to measure rotation speed, direction, position, angle, and length. For this purpose, the encoders convert mechanical movement into electrical signals. The incremental encoder is widely used in electric drives for estimating the speed and position. In the following case, the incremental encoder is used for an electromechanical device that generates pulses at outputs “A” and “B”, in response to incremental mechanical movements. There are two basic types of incremental encoders: linear incremental encoders, which detect linear motion, and linear rotary encoders, which detect the motion of a rotating shaft. In this paper, the rotary incremental encoder is used in combination with rotating structures represented by three-phase induction motors.

When an incremental encoder rotates at a constant speed, the output pulses take the form of squared waves. As the encoder rotates faster, the pulse rate increases accordingly. Because the relationship between pulse rate and speed is linear, it is a simple method of using pulse rate as a speed indicator. Furthermore, the output pulses can be converted into units of speed by measuring the output pulse’s frequency and multiplying it by an appropriate scale factor.

**Figure 7:**
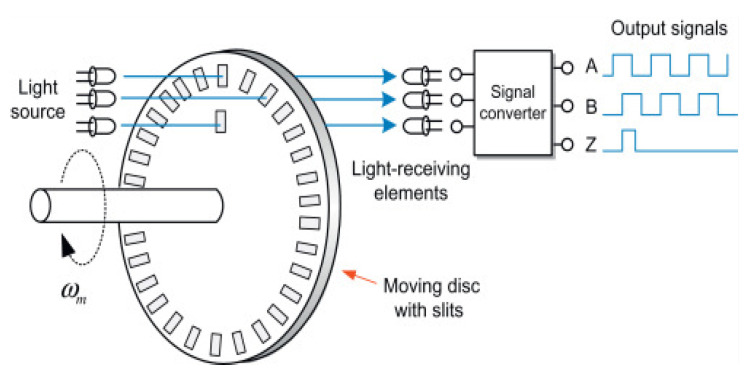
The operation of the rotary incremental encoder.

The resolution of the rotary incremental encoder is measured in pulses per rotation. The phase shift of two signals can be used to determine the direction of the shaft angle change. The encoders used here are placed at the ends of two induction motors that have an index channel named “Z”, which provides one pulse per revolution and verifies the output signals of “A” and “B” channels (Figure 7). These signals are the inputs of the microcontroller. The quadrature encoder pulses module of the microcontroller is used to evaluate the angular position by employing its hardware. Dedicated software for programming the microcontroller is needed.

The rotary incremental encoder is, in fact, a pulse generator consisting of a light source, a disk that rotates around a fixed axis, and a photodetector. The rotating disk has alternating opaque and transparent sections that either block or pass light to the photodetector. The detector receives light pulses as the disk rotates, emitting a stream of electrical pulses. The incremental encoder produces a sequence of three signals. The first two signals have a phase shift that is determined by the rotational direction. From these two signals, “A” and “B”, one can obtain the current rotor angular position. Signals create many pulses according to a position deviation if the position is changed (Figure 7).

**Figure 8:**
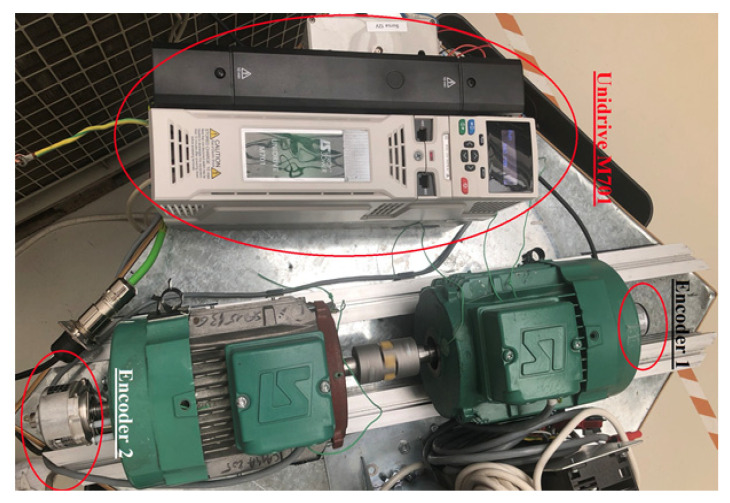
Photo of the experimental setup.

The proposed test bench consists of two induction motors coupled together, carrying identical incremental encoders; Figure 8. The power inverter Unidrive M701 is used for speed control of induction motors.

## 4. STM32 Nucleo F446RE Microcontroller

Frequency estimation is a high-performance speed prediction that requires dedicated hardware to provide encoder pulses and monitor the elapsed time interval. It is compatible with digital processing systems that have a fixed sampling timer.

The microcontrollers contain special peripheral devices named Quadrature Encoder Pulse modules. The rotary incremental encoder output “A” and “B” signals are used to increase or decrease a counter, to obtain the digital code of the rotor angular position. The module used in this paper is part of the STM32 microcontroller class.

The STM32F446xC device is based on an ARM Cortex-M4 core that operates at frequencies up to 180 MHz. The Cortex M4 core supports all ARM^®^ instructions and data types with single-data processing accuracy. It implements a set of DSP instructions and a memory protection unit, which improves application security. The STM32F446xC device incorporates embedded memories (flash memory up to 512 Kbytes, and up to 128 Kbytes of SRAM), up to 4 Kbytes of backup SRAM, and a wide range of enhanced and peripheral inputs/outputs connected to two APB buses, two AHB buses, and a 32-bit multi-AHB bus array. This device offers three 12-bit ADCs, two DACs, a low-power RTC, and twelve 16-bit general timers, including two PWM motor control timers and two 32-bit general-purpose timers.

**Figure 9:**
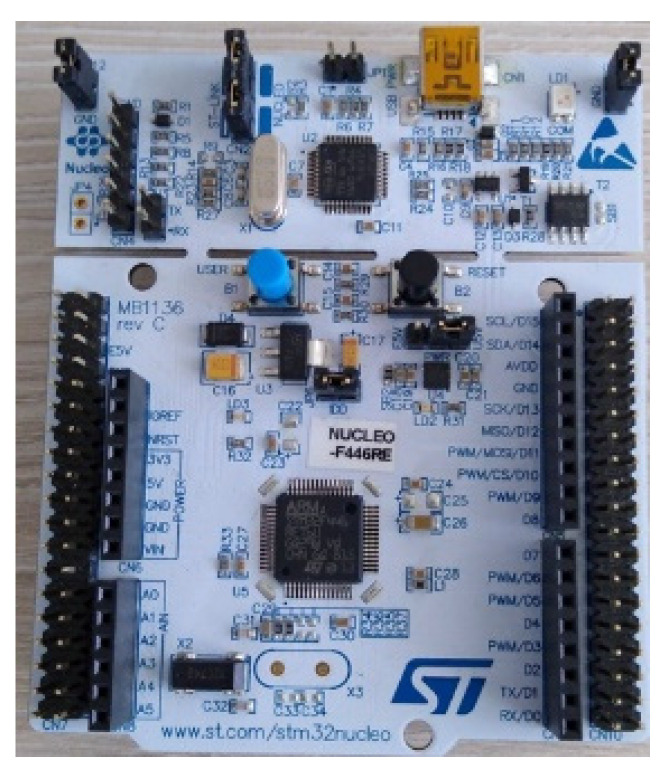
STM32 Nucleo F446RE microcontroller.

The STM32 Nucleo board (Figure 9) offers an accessible and flexible way for users to try new ideas and build prototypes with any line of STM32 microcontrollers, choosing from different combinations of performance, power consumption, and features.

### CubeMX Graphics Tool and the µVision IDE Programming Environment for STM32 Nucleo F446RE

First, to develop a program that reads the rotational speed of an incremental encoder and interprets it, both microcontroller configuration tool and programming environment are used to implement the necessary counter structure. Thus, the STM32CubeMX and programming medium µVision are employed to configure the microcontroller.

A brief description of the two programs and their functionalities is completed herewith. The software development environment used for STM32 is made in Keil (IDE). The graphical programming tool, STM32CubeMX, allows a very easy configuration of the STM32 microcontroller and initial C code generation for the Arm Cortex-M core. For microprocessors, the second step allows configuration of GPIOs and clocking for the entire system, as well as interactively assigning Arm Cortex-M peripherals. The tool provides a simple connection configuration with automatic conflict resolution.

**Figure 10:**
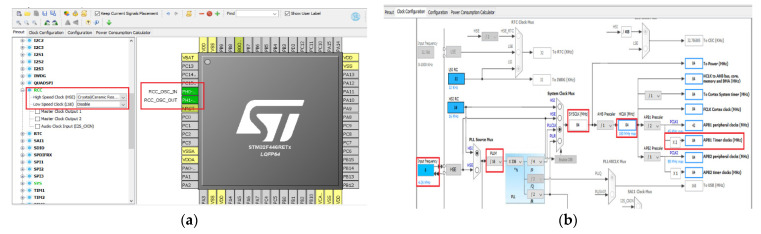
(**a**) Reset and control clock (RCC); (**b**) working frequency setup.

The first step is to select the STM32 development board or microprocessor that matches the set of peripherals, in this case STM32 Nucleo F446RE. Then, the input/output ports are assigned via RCC (reset and control clock); Figure 10a. Once the RCC is configured, one can move on setting the TIM3 timer from the Combined Channels and selecting the encoder operation mode. Thanks to the STM32CubeMx program, one can now set the clock values. The accuracy used is of 16 bits, and the working frequency is automatically set according to the given value HCLK (84MHz) (Figure 10b). The configurations are now saved in a file and the code is generated using the µVision5 Keil IDE software. Keil^®^ MDK is a powerful software development solution for Arm^®^ microcontrollers, and it has all the components needed to build and troubleshoot embedded applications.

**Figure 11:**
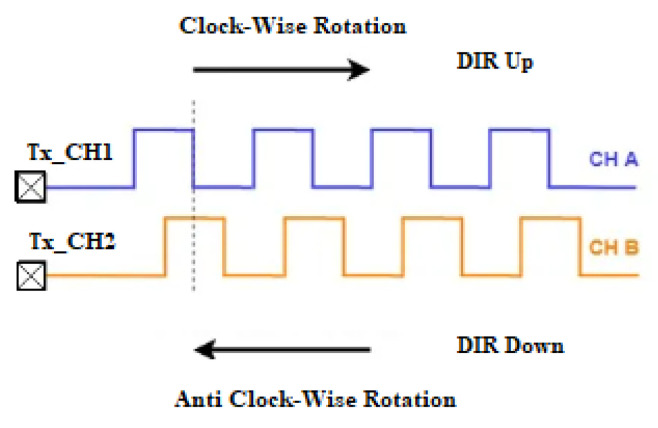
Recording the direction of rotation in the DIR register.

The two outputs, called A and B signals of the rotary incremental encoders, are called quadrature outputs, since they are 90 degrees out of phase, as shown in Figure 11. The direction of the motor depends on whether phase A drives phase B or vice versa. The third-channel Z index pulse occurs once per revolution, being used as a reference for measuring an absolute position.

A rotary encoder’s direction and position can be detected in a variety of methods. It is feasible to identify when the signal is HIGH and LOW by connecting pins A and B to two MCU inputs or outputs. It can be completed manually (using interruptions to capture when the channel changes state) or with the aid of a timer. The encoder’s channels can be configured in input acquisition mode, and acquisition data are compared to determine the encoder’s direction and speed. The STM32 has a simple mode for reading rotary encoders, called encoder mode, which simplifies the acquisition process. The direction of rotation is automatically calculated and stored in the TIMx DIR register, which is accessible to the programmer, Figure 11.

With the value obtained from the meter register, one can now find the number of RPMs using the number of pulses emitted by the encoder. Due to electromagnetic disturbances, the speed acquisition from the incremental encoder needs to be adjusted. One can use comparators as a filter bridge, especially if the encoder is in the proximity of power inverters or other noisy devices.

**Figure 12:**
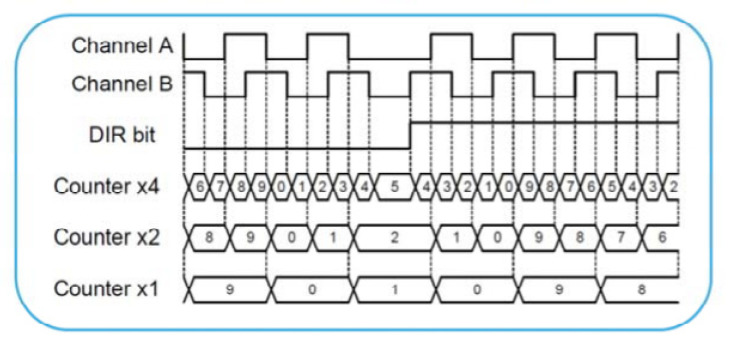
X1 encoding mode added.

The input filter stage of STM32 can be used to filter both channels A and B. The encoder mode is available for TI1, and TI2 and enables the HAL_TIM_Encoder_Init () function. The number of pulses is calculated automatically, and the direction of rotational movement is determined. The RPMs are derived for 1024 pulses for each revolution. Each timer embeds a 16-bit linear prescaler which allows division of the clock between 1 and 65,536. The counting pace can, therefore, be precisely adjusted. A division by 64 will result in a precise 1 MHz counting rate when the APB clock is 64 MHZ. An auto-reload register defines the counting period and an update event (interruption is issued on overflow). When the encoder interface is used, the STM32 timers implement a new counting mode. X1 mode is provided in addition to X2 and X4 modes, Figure 12.

In X1 mode, the counter value is updated on a single clock edge, as indicated by the timing diagram, depending on the direction. Here, when DIR is equal to 0, we use the falling edge of Channel A, and when DIR is equal to 1, we use the rising edge of Channel A. By moving from X4 to X2 to X1 mode quickly, the update interruption rate is often reduced. By adding the X1 encoding mode, the CPU burden is decreased for high RPM applications. Another advantage is that it supports all encoder modes (quadrature, clock plus direction, and directional clock). Two bits define in which state the index is detected. The index detection is obtained differently depending on counting direction to ensure symmetrical operation during speed reversal. The counter is reset during up-counting (DIR bit = 0) and the counter is set to TIMx_ARR when down counting.

Two sorts of encoder failures can be detected by the timing unit. Transition errors can be found in encoder systems. A state diagram can be used to depict the reading on the two inputs, which equates to a 2-bit gray code. It is predicted that only one bit will change at once. An incorrect transition may result in a transition error interruption. It is possible to identify faulty operation resulting in an excess of pulses per revolution for encoders with an index signal. A total of 4 × 1024 counts per revolution are produced by the encoder with 1024 pulses in each revolution. Each 4 × 1024 clock cycles, the index signal will reset the counter. Without an index event, if the counter value is increased from 0 to the auto-reload value or decreased from the auto-reload value to 0, an index position error will be reported. When this issue happens, an interruption may be generated.

**Figure 13:**
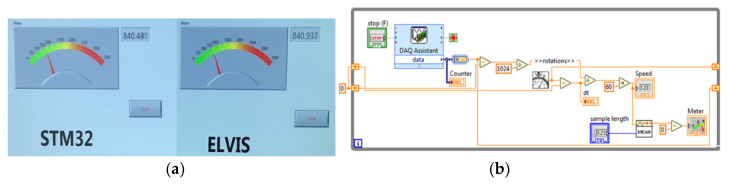
(**a**) The human–machine interface build in LabVIEW; (**b**) signal acquisition in NI-DAQmx.

Finally, the counter values, the clock value, and the value 0/1, depending on the direction of movement of the encoder, will be transmitted through the serial port to LabVIEW, and through the human–machine interface (HMI), the speed value is displayed; Figure 13a. To visualize the waveforms, one may use the NI ELVIS II instrument. For the signal, a logic diagram is made in LabVIEW—Figure 13b—for counting the pulses taken from the encoder through NI-DAQmx and the PFI3 digital counter input pin.

## 5. NI ELVIS II

**Figure 14:**
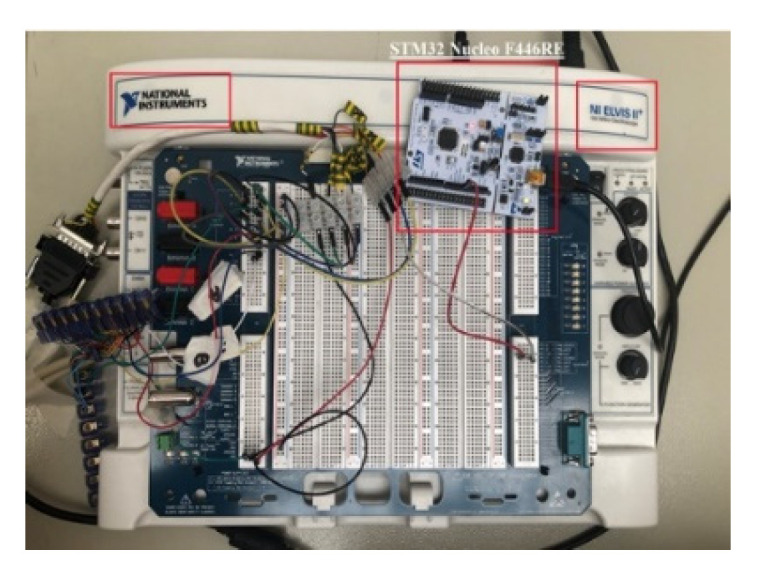
NI ELVIS II and STM32 Nucleo F446RE.

A valuable tool of those from National Instruments, NI ELVIS II, is also used in this work; Figure 14. It has very good built-in functionality to take the first steps in engineering and obtain tangible exposure to high-frequency measurement techniques. With this tool, you can acquire analog signals based on the built-in counter.

NI ELVIS II devices have 16 analog input channels. The first eight channels can be configured as a positive channel of a differential pair. If N is this channel, the N + 8 channel is the negative input of the pair. For example, if channel 1 is configured differentially, the positive input is channel 1 and channel 9 is the negative input. When creating a differential channel, only the physical channel name of the positive channel is used. NI ELVIS II equipment has two oscilloscopic physical channels, Dev1/scopeCh0 and Dev1/scopeCh1, available from the workstation.

NI ELVIS II devices have two physical analog output channels, two function generator channels, and two variable power channels. Function generator terminals have the same resource. NI ELVIS II devices have 24 digital input and output lines in terms of digital ports. These lines belong to a single port. Port 0 can perform static digital operations. NI ELVIS II devices have two more ports, port 1 and port 2. Port 1 has eight digital I/O lines. Port 2 has seven digital I/O lines. NI ELVIS II devices have two counters/timers. There are four primary terminals used as SOURCE, GATE, AUX, and OUT functions. NI-DAQmx has default values for these terminals.

## 6. Experimental Results and Conclusions

The first purpose of this paper is to identify and monitor the differences between the speed values acquired from the three devices, through the development boards STM32 Nucleo F446RE [18], NI ELVIS II, and the Unidrive M701 power converter, respectively. On the one hand, with the aid of the frequency power converter connected to the induction motor and the two encoders, one enters different frequency values to reach a predefined speed on the control console.

On the other hand, one reads the speed values on the three devices, i.e., the STM32 Nucleo development board through a counter algorithm made in the µVision programming environment, the equipment from National Instruments ELVIS II using a configuration in the LabVIEW environment, and, finally, the Unidrive M701 power converter.

The experimental measurements performed using this equipment are provided. Hence, the equipment used consists of two encoders with 1024 PPR, two induction motors, the Unidrive M701 frequency converter, the STM32 Nucleo F446RE development board, and NI ELVIS II. One reads the speed values with all three devices at once. During the measurements, all the three pieces of equipment behaved very similarly for the speed ranges (0–1500 rpm). However, sensitivity to electromagnetic disturbances of the STM32 device was revealed, which is due to the low acquisition cost and prototyping conditions of the development board.

From a technical point of view, the STM32 Nucleo system has one of the best available purchase prices. Regarding the STM32 Nucleo F446RE microcontroller, the board is configured with the STM32 CubeMx instrument and programmed using µVision software, to calculate the counter. The obtained values are transmitted through the serial port to LabVIEW; afterwards, the speed is displayed according to the received pulses.

For ELVIS II, one uses the digital input pins, by which the pulses are sent to the program and then converted to rpm and showed on the HMI interface. The sampling frequency used was 100 kHz, and the time period between two points was T = 100 µs.

**Figure 15:**
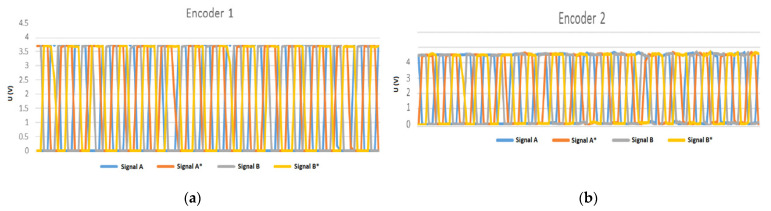
(**a**) A and A*, and B and B* signals for incremental encoder 1; (**b**) A and A*, and B and B* signals for incremental encoder 2.

**Figure 16:**
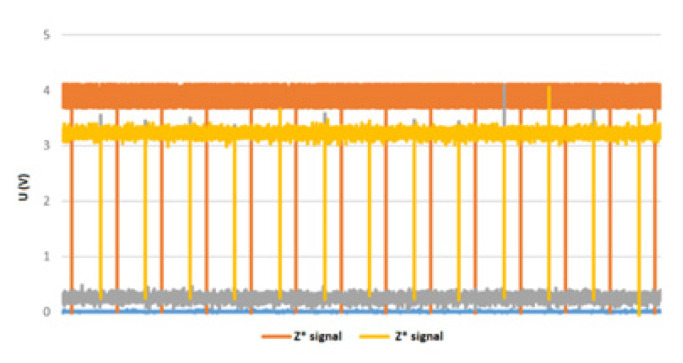
Z and Z* signals for incremental encoders 1 and 2.

The experiment was performed with the following technical specifications: Randomly taken, speed = 804 rpm; Figure 9. Sampling frequency = 100 kHz. The waveforms corresponding to the signals A, A*, B, and B*, are shown in Figure 15a for encoder 1, and signals A, A*, B, and B* are shown in Figure 15b for encoder 2. The encoder 1 is connected to the STM32 device and encoder 2 is connected to NI ELVIS. The signals A, A*, B, and B*, respectively, are out-of-phase by 180 degrees each, and signals A and B are 90 degrees out-of-phase. The pulses under the same conditions were also acquired, as well as for Z and Z* signals (Figure 16) of the two encoders. It can be pointed out that speed acquisition from an incremental encoder is feasible using the STM32 Nucleo system, and further improvement of the processing capacity inside the Unidrive M701 power converter is possible for a high-performance and cost-effective solution.

Moreover, the acquired values and waveforms of the signals transmitted by the two incremental encoders are in good agreement. The differences between experimental samples are very small or even non-existent, thus confirming the initial assumption of the present study. The original step-by-step programming methods were carried out for the STM32 microcontroller and NI ELVIS. Programming methods in C++ and LabVIEW were developed and tested along with hardware implementation and configuration. This research work conducted over several years can provide guidelines for incremental encoder speed acquisition’s future developments.

The main benefits of the proposed and experimentally tested method are related to its low cost, low power consumption, and straightforward speed acquisition implementation, as compared to FPGA, where more complex hardware and software strategies need to be implemented. Compared with the GLMT, where the use of FPGA is needed, it is known that configuration of an FPGA is difficult since the designer must deal with a high level of programming complexity. FPGA tends to use more power than a microcontroller or an ASIC because not all blocks can be used when creating an application. FPGA can be expensive for a straightforward application. There is no internal oscillator; hence, an external source of a clock must be given for the FPGA. The MT approach’s disadvantage is that it necessitates a lot of computing resources. Similar to the MT type, the DLMT algorithm performs similarly, but not at low speeds when noticeable oscillations are found. In this application, the STM32 is preferred when designing the actual system with minimal hardware requirements, and it is set to perform specific functions such as speed acquisition. Angular speed and acceleration can be obtained as continuous signals and can be acquired for low, medium, and high speeds. Also, by adding the X1 encoding mode, the CPU burden is decreased for higher RPM applications. A number of STM32 devices and evaluation boards are supported thanks to a collaboration between MathWorks and STMicroelectronics. Therefore, it is easy to develop, load, and run algorithms on STM32 devices using Simulink models with the help of the Embedded Coder Support for STMicroelectronics Discovery Boards (with new STM32CubeMX support for all STM32F4x devices) and Simulink Coder Support for Nucleo Boards.

## Data Availability

Not applicable.
